# Summer primary production of Arctic kelp communities is more affected by duration than magnitude of simulated marine heatwaves

**DOI:** 10.1002/ece3.70183

**Published:** 2024-09-29

**Authors:** Cale A. Miller, Frédéric Gazeau, Anaïs Lebrun, Samir Alliouane, Pierre Urrutti, Robert W. Schlegel, Jean‐Pierre Gattuso, Steeve Comeau

**Affiliations:** ^1^ CNRS, Laboratoire d'Océanographie de Villefranche Sorbonne Université Villefranche‐sur‐Mer France; ^2^ Institute for Sustainable Development and International Relations, Sciences Po Paris France; ^3^ Present address: Department of Earth Sciences Geosciences, Utrecht University Utrecht The Netherlands

**Keywords:** Arctic, kelp, marine heatwave, net community production

## Abstract

Fjord systems in the Norwegian Arctic are experiencing an increasing frequency and magnitude of marine heatwaves. These episodic heat stress events can have varying degrees of acute impacts on primary production and nutrient uptake of mixed kelp communities, as well as modifying the biogeochemical cycling in nearshore systems where vast areas of kelp create structural habitat. To assess the impact of future marine heatwaves on kelp communities, we conducted a 23 day mesocosm experiment exposing mixed kelp communities to warming and heatwave scenarios projected for the year 2100. Three treatments were considered: a constant warming (+1.8°C from the control), a medium magnitude and long duration heatwave event (+2.8°C from the control for 13 days), and two short‐term, more intense, heatwaves(5 day long scenarios with temperature peaks at +3.9°C from the control). The results show that both marine heatwave treatments reduced net community production, whereas the constant warm temperature treatment displayed no difference from the control. The long marine heatwave scenario resulted in reduced accumulated net community production, indicating that prolonged exposure had a greater severity than two high magnitude, short‐term heatwave events. We estimated an 11°C temperature threshold at which negative effects to primary production appeared present. We highlight that marine heatwaves can induce sublethal effects on kelp communities by depressing net community production. These results are placed in the context of potential physiological resilience of kelp communities and implications of reduced net community production to future Arctic fjord environmental conditions.

## Graphical abstract

This study aims to understand the potential impacts of marine heatwaves now and in the future on Arctic kelp ecosystems by simulating scenarios projected for the year 2100. Our findings reveal that both marine heatwave treatments significantly reduced net community production. Notably, the long marine heatwave scenario had a more severe impact on accumulated net production compared with the two short‐term heatwaves.

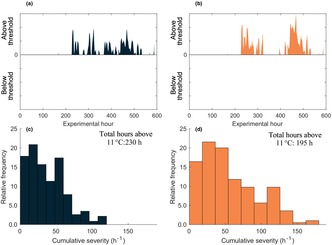

## 1 INTRODUCTION

The structure and function of European Arctic fjord ecosystems are rapidly encountering multiple perturbations that affect their chemical and physical environments, brought on by a warming climate (Konik et al., 2021; Schlegel et al., 2023). These changes are manifest as sea ice and glacial retreat, warming waters, increased turbidity and sedimentation, and freshening of Arctic fjords. Although long‐term time series records afford insight to the precipitously changing physicochemical environment, predicting the biological response to these drivers remains challenging (Bloshkina et al., 2021; Geyman et al., 2022; Schlegel & Gattuso, 2023; Węsławski et al., 2011). This difficulty obscures the ability to understand the future function and structure of Arctic fjords as they undergo the process of borealization—a transformation of Arctic‐type ecosystems to that of subarctic with an associated poleward shift of marine species (Fossheim et al., 2015; Ingvaldsen et al., 2021; Polyakov et al., 2020). The degree to which borealization may affect the biogeochemical cycling, biodiversity, and organismal tolerance in Arctic fjords will depend on multiple drivers (e.g., water circulation, organic carbon sedimentation rates, and human activity) inducing physicochemical changes (Kujawa et al., 2021). To that effect, comparing Arctic fjords with lower latitude fjords, or adjacent fjords with different physicochemical characteristics (i.e., natural analogue systems), may provide evidence of future Arctic fjord conditions (Kujawa et al., 2021; Węsławski et al., 2017). In fact, many studies have shown that the borealization process in Arctic fjords has led to a restructuring of pelagic and benthic communities that stimulate an increased resilience and maturation of biological diversity and organization (Frainer et al., 2021; Ingvaldsen et al., 2021; Paar et al., 2019; Węsławski et al., 2011, 2017).

The warming of European Arctic fjords is not linear, and is susceptible to land heatwaves and warming pulses that occur with the intrusion of North Atlantic waters (Ingvaldsen et al., 2021). Thus, warming anomalies, or marine heatwaves (MHW), in the Arctic are occurring at an accelerated pace and are propagated by North Atlantic warming anomalies. These MHWs are defined as anomalous temperature events that exceed 5 days with temperatures warmer than the 90th percentile of observations using a 30 year historical baseline (Hobday et al., 2016). With respect to the Barents Sea, the annual mean frequency and duration of MHWs has increased by 62% and 31%, respectively, over the past two decades (Mohamed et al., 2022). The effects of MHWs on benthic organisms in Arctic fjords suggest potential susceptibility to these warming anomalies (Jordà‐Molina et al., 2023). However, the restructuring of benthic communities by non‐native species in response to frequent or chronic environmental changes may provide some resilience to MHWs (Węsławski et al., 2017; Ingvaldsen et al., 2021 and references therein; Goldsmit et al., 2024). Relevant in this context is the response of biogenic habitats created by large brown benthic macroalgae (i.e., kelp), which play a crucial role in supporting benthic ecosystems on rocky nearshore coasts and fjords (Christie et al., 2009; Włodarska‐Kowalczuk et al., 2009). Although it appears that Arctic kelp species may be tolerant to high temperatures under a future climate, more work is needed to understand how kelp communities will respond with respect to physiological resilience and community production to frequent and intense warming anomalies occurring in the Arctic (Filbee‐Dexter et al., 2019 and references therein; Miller et al., 2024a).

It is well documented that kelp throughout temperate latitudes are susceptible to MHWs, which has resulted in large‐scale die‐offs and a retraction of growth (Filbee‐Dexter et al., 2020; Smale, 2020; Wernberg et al., 2016). The physiological tolerance of kelp to MHWs is marked by their ability to acclimate to a range of specific temperature regimes across a latitudinal gradient (Andersen et al., 2013; Hollarsmith et al., 2020). Although the role of thermal niches in determining organismal biogeographical zones and ecological processes has been long established (Hutchins, 1947), the ability of kelp populations to tolerate, recover, and adapt to anomalously high temperatures may be insufficient in a future climate. This susceptibility could lead to increased fatality within these kelp populations when facing MHWs (Filbee‐Dexter et al., 2020).

Kelp species present in the Arctic such as the genera *Saccharina* and *Laminaria* display a broad range of temperature optima and thermal tolerance (Bolton & Lüning, 1982; Davison, 1987; Davison et al., 1991). This physiological tolerance is demonstrated by similar rates of photosynthesis observed across temperature levels ranging from 0 to 20°C, as well as a wide thermal optimum for growth spanning 5°C to nearly 20°C for *S. latissima* (Davison, 1987; Davison & Davison, 1987; Lebrun et al., 2022 and references therein). Similarly, physiological tolerance to thermal stress has been observed for sporophytes of *L. digitata* (0–23°C) and *S. latissima* (to 5–20°C), with necroses finally setting in at temperature levels above 20°C (Andersen et al., 2013; Bolton & Lüning, 1982; Karsten, 2007; Liesner et al., 2020). Although these scopes of thermal tolerance show kelp resilience to temperature, their reaction to stochastic MHWs is less clear. This observation was evidenced by declines in photosynthetic efficiency when kelp were exposed to high or low irradiance in combination with MHWs, highlighting the combined effect of light stress with temperature (Bass et al., 2023; Niedzwiedz et al., 2024). Although these studies highlight the limits of tolerance to MHWs from a physiological perspective for acclimated ecotypes, investigating the effects at the community level can provide a clearer perspective of the functioning and structure of future Arctic kelp communities.

This study used a mesocosm approach to investigate the net community production and potential tolerance of mixed kelp communities (with associated fauna) living in lower latitude Arctic fjords to MHWs. Previous studies have shown kelp to be susceptible to the intensity of MHWs in more temperate latitudes (Filbee‐Dexter et al., 2020), however, this study examines not just intensity, but the duration and recovery potential of Arctic kelp to MHWs. Although kelp species appear to exhibit a robust tolerance to thermal stress, the scope of resilience is defined by ecotype and regional acclimation (Diehl et al., 2021; King et al., 2019). This study hypothesized that (1) the metabolic response and production of mixed sporophyte kelp assemblages would differ according to the frequency, magnitude, and duration of MHW events, and (2) the effects of MHWs would manifest as a decrease in survival and net community production. The importance of understanding kelp community production under differing MHW scenarios provides insight into biogeochemical cycling as well as benthic production and structure. As a crucial habitat in nearshore systems, we present evidence for kelp community production under differing MHW frequencies and magnitudes and conclude that, low magnitude, long duration events appear more stressful than short‐term, high magnitude events and frequencies.

## 2 METHODS

### 2.1 Sampling sites

Kelp populations of *Saccharina* and *Laminaria* along Norway's mid‐ and high‐latitude (65–71° N) rocky coasts have been reduced on the order of millions of tons of biomass by overgrazing sea urchin populations—first observed in the late 1970s and 1980s (Christie, Gundersen, et al., 2019; Norderhaug & Christie, 2009; Sivertsen, 2006). Kelp populations in the most southern parts of this range have recovered over the past 15 years. However, this recovery has been slower in the north around the area of Troms (69.0–69.8° N) and Finnmark (>70° N), where urchin barrens are particularly persistent (Christie, Gundersen, et al., 2019). For this study, the region of Troms was chosen as an area that represents a low‐latitude Arctic fjord system (R. W. Schlegel & Gattuso, 2023), acting as a natural analogue of a high‐latitude fjord system (Kujawa et al., 2021).

Kelp community members (sporophyte kelp and macrofauna) were identified (H. Hop, pers. comm.) throughout the Troms region and collected at three different sites: Melhomen (69.88° N, 18.86° E), Sommarøy (69.63° N, 17.97° E) and Kvaløyvågen (69.85° N, 18.82° E). Temperature patterns were similar across the sampling locations where the median value ranged from 8.5 to 11.4°C from mid‐June to mid‐July 2022 (Figure S1). The maximum distance between sampling sites was ~44 km (between Melhomen and Sommarøy). The three kelp species identified for collection were *Alaria esculenta*, *Laminaria digitata*, and *Saccharina latissima*. All species were found to be cohabiting at each collection site, however at different densities. To reduce the impact of oversampling in one location, mature sporophytes and a mix of benthic fauna (snails, mussels, and urchins: see details in Section 2.2) were sampled across the three sites via scuba diving to depths of 1–7 m during the last 2 weeks in June 2022. The proportion of kelp sporophyte and fauna samples collected at each site was ~37%, 33%, and 30% at Melhomen, Kvaløyvågen, and Sommarøy, respectively. The depth distribution of kelp was limited to shallow regions (2 m in protected sites and 5–10 m for more exposed sites) due to excessive urchin grazing in the region. The tidal range at the sampling sites ranged from 2.5 to 3 m. Summer stratification occurs primarily in June, whereas in the spring season, stratification is usually weak and can be broken by strong winds; however, this depends on the amount of freshwater run‐off which varies year‐to‐year and the width of the fjord (Wassmann et al., 1996).

### 2.2 Experimental setup

Twelve 1 m^3^ (~1.2 m in height and a mean diameter of 1.1 m) circular mesocosms made from fiberglass were installed on the outdoor premises (i.e., an open and paved area clear from building shadows) within the center of aquaculture station Havbruksstasjonen i Tromsø (Kårvik, Norway; 69.9° N, 18.8° E) for a three‐week MHW exposure experiment on kelp communities. Community assemblages—kelp and macrofauna—were reconstructed in each mesocosm based on densities and average biomass values reported for Arctic coasts between 1.5 and 7 m depth (Hop et al., 2012; Paar et al., 2016). Each mesocosm was stocked with 2–8 individual sporophytes of each kelp species, ranging between 43 and 188 cm in length. The kelp were evenly distributed to achieve a total fresh weight (fw) biomass per species of 1000 g for *A. esculenta*, 500 g for *S. latissima*, and 1000 g for *L. digitata*. Kelp biomass in each mesocosm was measured at the beginning (*T*_0_) and again at the end (*T*_F_) of the experiment. The target biomass for selected fauna were based on values reported in other Arctic fjords (Paar et al., 2016). Selection of fauna was determined by observed species abundance (i.e., these organisms appeared in abundance at all sampling locations) at each sampling location by the dive team. Sea urchin (*Strongylocentrotus droebachiensis*) total biomass per mesocosm was between 250 and 280 g fw. Urchins were placed in a 30 cm^3^ wire mesh cage suspended in each mesocosm to prevent feeding on the kelp in each tank. Scraps of kelp from excess individuals not used for the experiment weighing <10 g in fw were placed in the cages for urchin satiation and replaced when needed. Mussels (*Modiolus modiolus*) and gastropods (*Neptunea despecta*) were placed directly in the mesocosm for a total mass of 415 g and 248 g per mesocosm, respectively. Four to six rhodoliths (coralline algae) per mesocosm measuring three to eight cm in length of substrate cover on oval‐shaped rocks were distributed as evenly as possible across mesocosms. Further information concerning the response of faunal groups and individual kelp species to the experimental conditions can be found elsewhere (Lebrun, A., Miller, C.A., Gazeau, F., Urrutti, P., Alliouane, S., Gattuso, J‐P., Comeau, S., unpublished data).

The experimental design consisted of four conditions: a control (ambient seawater), a constant high temperature treatment (HT), a long duration and low amplitude heatwave treatment (1MH), and a high frequency and high magnitude heatwave treatment (2MH). Each condition was replicated in triplicate totaling 12 mesocosms, which were haphazardly distributed in two rows. The rows were spaced 2 m apart across an 18 m^2^ area, where each mesocosm within a row (6 mesocosms per row) was spaced ~0.5 m apart. Replication was limited to triplicates for each treatment due to the size of the mesocosms and the ability to manipulate temperature appropriately while maintaining consistent flow rates. Each mesocosm was supplied with flowing seawater that was directly pumped from a depth of 30 m in front of the Havbruksstasjonen i Tromsø station. Incoming seawater was first stored in a retention basin from where it was pumped to each mesocosm using a submersible pump (Albatrosⓒ, Norsk Pumpeservice AS). A variation of the automated temperature perturbation system described in Miller, Urrutti, et al. (2024b) was integrated with a single heat pump that warmed ambient seawater to 15°C which was subsequently mixed with ambient seawater to achieve targeted temperature levels. The automated flow valves of the system, regulated by communication feedback from continuous measurements of temperature taken inside each mesocosm, mixed precise volumetric proportions of heated and ambient seawater to a single intake port fixed to each mesocosm at a rate of 7–8 L min^−1^. Turnover time in each mesocosm was ~2 h.

In each mesocosm, oxygen (O_2_), temperature, and salinity were measured at high frequency (one measurement per minute) using an in situ optical O_2_ sensor (Aqualabo©, PODOC) and a temperature‐conductivity probe (Aqualabo©, PC4E). A 12 W wave pump (Sunsunⓒ JVP‐132, flow rate = 8 m^3^ h^−1^) was fixed inside each mesocosm to ensure a well‐mixed water column, and water flow for the kelp. Photosynthetically available radiation (PAR) loggers (Odysseyⓒ) were centered in each mesocosm (~5 cm below the surface) and fixed to a straight piece of polyvinyl chloride (PVC) tube that was securely attached to the bottom. Mesocosms were covered with circular acrylic lids equipped with a green (RL244) and neutral light filter (RL211; Lee Filtersⓒ, LA‐BS) that replicated the underwater light attenuation and spectrum at 5 m depth.

### 2.3 Experimental design

**FIGURE 1:**
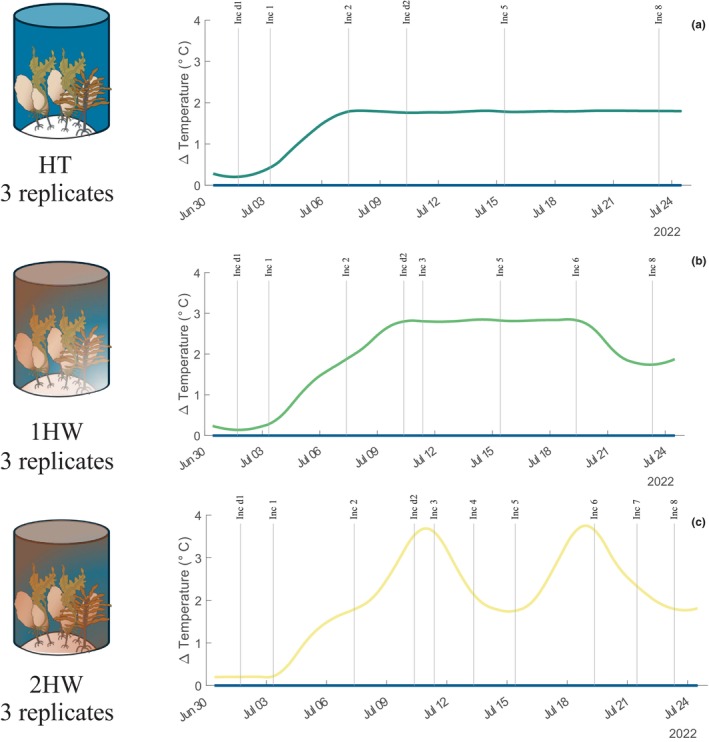
Experimental conditions for treatments: (a) Constant high temperature (HT), (b) one marine heatwave (1MH), and (c) two marine heatwaves (2MH), as offsets from the control treatment which reflected ambient temperature.

The quantification and assessment of kelp community production in response to differing heatwave scenarios and effects through time began on 2022‐06‐30, and was terminated on 2022‐07‐23. Of the four different conditions, the control treatment tracked in situ temperature, and the HT treatment was maintained at a consistent offset of ~1.8°C above ambient (i.e., control) temperature (Figure 1a). This offset value was determined by extrapolating the projected 2100 sea surface temperature (SST) in the region at the current rate observed over the last 40 years (+ 0.22°C per decade). This was based on NOAA's long‐term climate data record (daily Optimum Interpolation Sea Surface Temperature; OISST). The 1MH treatment had a peak magnitude offset from ambient of +2.8°C for a duration of 13 days (Figure 1b). The 2MH treatment exhibited two high‐magnitude peaks lasting 5 days and reaching +3.9°C above ambient temperature. These peaks were separated by a period of 3 days and followed a slow ramp‐up and ramp‐down incremental change from the HT condition (Figure 1c). The magnitude of the two heatwave treatments were applied as offset values from the predicted 2100 SST with a duration and peak that aligned well with this region assessed using https://www.marineheatwaves.org/tracker.html (Schlegel, 2020).

All mesocosms were kept at ambient conditions for the first 48 h before increasing temperature by 0.58°C over 3 days to reach +1.8°C in the warming and two heatwave treatments. Temperatures for the two heatwave treatments increased from that point 48 h later (Figure 1 and Figure S2).

Net community production (NCP) for each treatment was quantified by performing closed mesocosm incubations. Incubations were performed weekly for the control and HT condition, and at set time points for the simulated heatwave treatments (1MH and 2MH) to capture the response of NCP at the peak of a temperature anomoly, and again on the return from peak conditions (Figure 1b,c). Incubations were performed by completely filling each mesocosm to the rim by closing the outflow valve. Once overflowing, incoming water was turned off for a period of 3 h before returning flow and opening the outflow valve. All sensors in each mesocosm recorded dissolved O_2_, salinity, and temperature every minute, PAR was recorded every 10 min. All incubations were performed mid‐morning for consistency. Community respiration was measured twice during the experiment by following the same incubation procedure as above, but was performed by covering the top of each mesocosm with three layers of black plastic films. Every mesocosm was cleaned regularly by brushing the walls of epiphytes. Each sensor (i.e., O_2_, PAR logger, and temperature‐conductivity probe) was cleaned every two to three days, and again directly before performing an incubation. The calibration for the O_2_ sensor was performed prior to the start of the experiment, but on site, using a two‐point calibration at 0% and 100% saturation. For both the temperature‐conductivity sensor and the PAR logger, a single point offset calibration was applied using reference measurements by a Sea‐Bird SBE37 CTD, and an underwater quantum LI‐COR (model 192) sensor, respectively. Calibration was performed at the same time as the O_2_ sensor.

### 2.4 Quantitative and statistical methods

Rates of NCP were calculated for each incubation as the change in O_2_ over an hour‐long period (i.e., 3 hourly rates for every 3 h incubation) using a least squares linear regression. NCP and community respiration (CR) rates were normalized to the m^2^ footprint of each mesocosm. Temperature, salinity, and PAR measurements were filtered to remove erroneous values using the isoutlier function in Matlab (V2023b) and setting a percentile threshold of 0.001 and 0.995 for temperature and salinity, and 1.2 times the inter‐quartile range (IQR) for PAR.

Treatment (predictor variable) effects on NCP rates (dependent variable) were compared using a stepwise linear model to assess across treatment differences. Between treatment comparisons were performed using a contrast matrix to compare model estimated coefficients for the significant predictor variables. Both heatwave treatments (i.e., 1MH and 2MH) and both dark respiration incubations were scrutinized for a time effect on NCP and CR rates using repeated measures ANOVA. Changes in kelp biomass from T_0_ to T_F_ were compared across treatments with a 1‐way ANOVA.

Calculated NCP rates were aggregated and sorted by temperature irrespective of treatment to derive temperature dependent photosynthesis‐irradiance (P‐I) curves. Timepoints (i.e., date and time of an incubation) where temperature was similar across treatments were collated and binned by a window of ~1°C (except for the extreme temperature step which was 0.5°C) using a hyperbolic tangent model (Table 1). Robust model fits were used to determine P–I model coefficients: maximum photosynthetic rate (*P*_max_), the photosynthetic efficiency (*α*), and the compensation irradiance point (*I*_c_).

**TABLE 1:** Model results of net community production predicted using a hyperbolic tangent model for group temperature scenarios pooled from across all treatment conditions.

Parameter	Coefficients estimate	SE	*t*Stat	*p‐*Value
Model: Low temperature (7.5–8.5°C)				
*P*_max_	26.58	2.070	12.84	<.001
Alpha	0.551	0.127	4.350	<.001
CR	−5.049	2.290	−2.205	.046
Observations (*n*)	34			
RMSE	2.62			
Model: Medium temperature (10.1–11.1°C)				
*P*_max_	27.50	2.34	11.74	<.001
Alpha	0.307	0.073	4.223	<.001
CR	−8.11	2.462	−3.292	.002
Observations (*n*)	76			
RMSE	2.67			
Model: High temperature (11.5–12.6°C)				
*P*_max_	17.89	2.303	7.771	<.001
Alpha	0.303	0.108	2.810	.009
CR	−8.34	2.612	−3.194	.004
Observations (*n*)	29			
RMSE	2.66			
Model: Extreme temperature (13.0–13.5°C)				
*P*_max_	9.07	1.040	8.719	<.001
Alpha	0.091	0.0176	5.132	.001
Observations (*n*)	9			
RMSE	1.06			

Abbreviation: CR, community respiration.

An aggregated temperature dependent model was derived by incorporating the negative effect of temperature on *P*_max_ and α into a modified hyperbolic tangent model that was used to estimate NCP as:

(1) $$\mathrm{NCP}=P_{\max}-T\times \tanh\left(\frac{\mathrm{\alpha I}-T}{P_{\max}}\right)+R_{d},$$

where *P*_max_ is the maximum NCP (mmol O_2_ m^−2^ h^−1^), *I* is the PAR (mmol photons m^−2^ h^−1^), *α* is the initial slope of the curve (mmol O_2_ m^−2^ h^−1^(mmol photons m^−2^ h^−1^)^−1^), *R*_d_ is the dark respiration rate (mmol O_2_ m^−2^ h^−1^), and *T* is the temperature in°C. The partial dependency between the two predictor variables in the model, PAR and temperature, were examined as it relates to their individual effect on NCP rates. Accumulated net community production was estimated for a 3‐week period by summing all predicted hourly rates (positive rates only) from the model output using the predictor variables of PAR and temperature for the control, 1MH and 2MH treatments. Only positive rates were considered due to the fit of the model (see Section 3.4). A Monte Carlo simulation of 1000 iterations was performed to quantify the error in estimated accumulated net community production by sampling values within the model predicted 95% CI for the control, 1MH and 2MH treatments.

Differences between the 1MH and 2MH treatments were compared by quantifying the number of hours and magnitude above an 11°C threshold (value chosen based on analysis of temperature effects on predicted model coefficients *P*_max_ and *α*). A cumulative severity index was calculated as:

(2) $$\text{Cumulative severity}=\frac{\sum_{i=1}^{n}\left(x_{i}-\text{Thresold}\right)\;}{D},$$

where *x*_i_ is the temperature above the *Threshold* (11°C), and *D* is the duration in hours.

## 3 RESULTS

### 3.1 System performance and conditions

Over the 3‐week experiment, four incubations were conducted on the control and HT treatment, six incubations for the 1MH treatment, and eight incubations for the 2MH treatment. Not all treatments were incubated at every incubation timepoint due to the co‐occurrence of sampling individual organisms in specific treatment mesocosms during the community incubations (Lebrun, A., Miller, C.A., Gazeau, F., Urrutti, P., Alliouane, S., Gattuso, J‐P., Comeau, S., unpublished data). The temperature in each of the treatment mesocosms was successfully regulated over the experimental period as deviations were held below <0.5°C for 94% of the time (Miller, Urrutti, et al., 2024b). The standard deviation (SD) of temperature during incubations—where flow was shutoff for a period of 3 h—across replicates and treatments was <0.13°C. The range of the average temperature increase across all treatments during incubations was <0.88°C.

**FIGURE 2:**
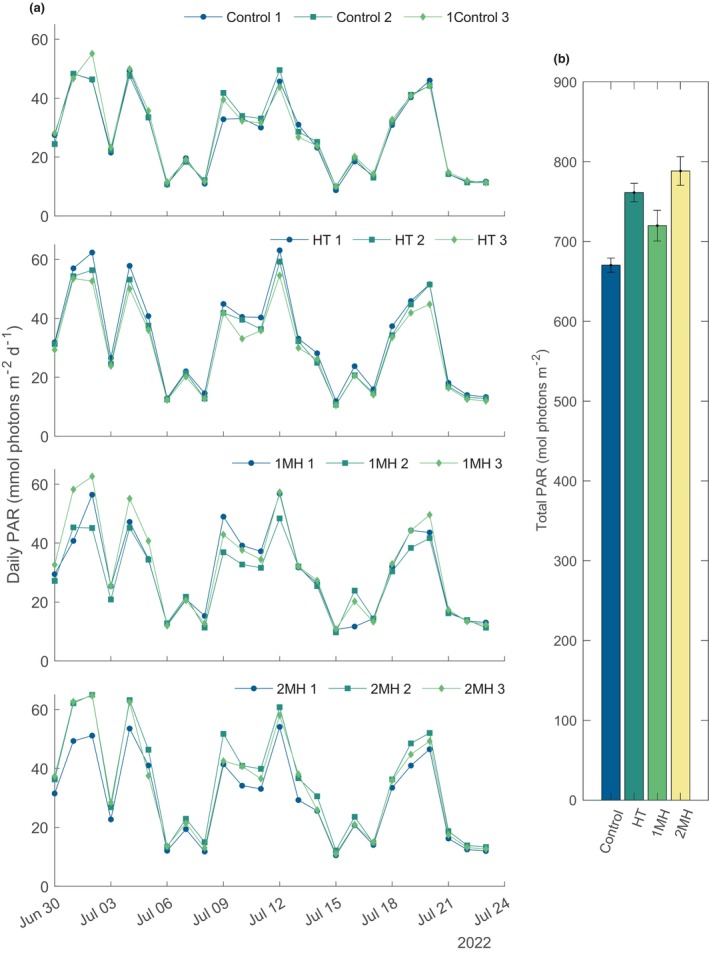
(a) Daily integrated photosynthetically available radiation (PAR) for each treatment. Numbers 1–3 in the legend refer to a replicate. (b) Total integrated PAR for the entire experimental period as the mean of the summed measurements for each treatment. The error bars are the propogated SD of the three replicates per treatment.

The daily integrated irradiance in the mesocosms ranged between 14 and 65 mol photons m^−2^ d^−1^ with minimal variation across replicates with an average SD that ranged from 1.32 to 2.99 mol photons m^−2^ d^−1^ (Figure 2). The random placement of replicates across the ~18 m^2^ area resulted in the variance of irradiance flux received. The PAR logger in the 3rd replicate of the 2MH mesocosm started recording erroneous values on 18‐Jul‐2022 03:50:00 (UTC). For the remaining 5 days of the experiment, the average of the two other replicate mesocosms were used for determining PAR during the final incubations and daily integrated irradiance. During this period, the range of absolute difference between the two replicates used to average the third replicate value was <2 mmol photons m^−2^ h^−1^.

Fresh weight biomass at the beginning of the experiment was not significantly different across treatments; however, final kelp fw for the 1MH treatment was significantly lower compared to the control (*F*_3,8_ = 6.26, *p*‐value = .0171; Figure S3). For the control and HT treatments, kelp fw increased for all replicates, whereas biomass decreased for 2 out of the 3 replicates in the 2MH, and in all replicates for the 1MH treatment (Figure S4).

### 3.2 Net community production and community respiration

**FIGURE 3:**
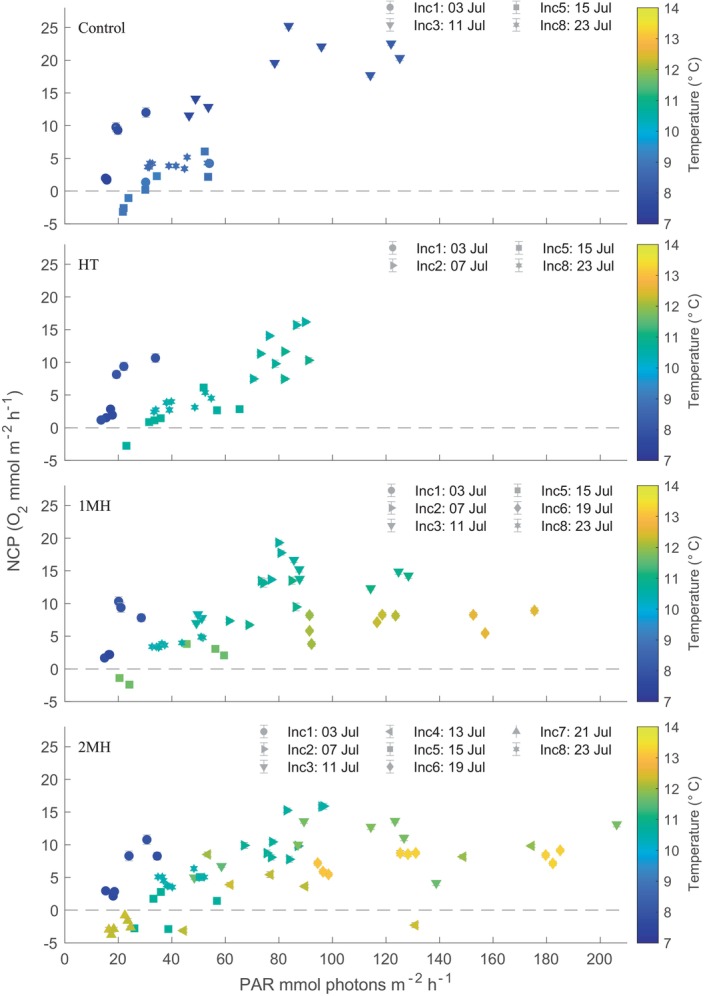
Net community production (NCP) rates for each treatment condition as a function of photosynthetically available radiation (PAR) where the colored markers reflect the average temperature during the incubation. Symbols correspond to the different incubations and the date is included in the legend. Note that not all treatments were incubated at the same time (see Figure 1). Error bars are the SE of the linear model rate estimate.

The NCP rates were highest for the control treatment, however, only the 1MH and 2MH treatments were significantly different compared to the control (Table S1). Both temperature and PAR were significant predictor variables (*p*‐value < .001) for NCP rate. In the control and HT treatment, temperature never exceeded 11°C, whereas temperature peaked at 12.5°C for the 1MH treatment and 13.3°C for the 2MH treatment (Figure 3). Incoming PAR only exceeded 100 mmol photons m^−2^ h^−1^ during incubations 3, 4 and 7. Across all treatments, NCP was held below 13.5 mmol O_2_ m^−2^ h^−1^ whenever temperature was >12°C while it went up to 25 mmol O_2_ m^−2^ h^−1^ when <12°C.

**FIGURE 4:**
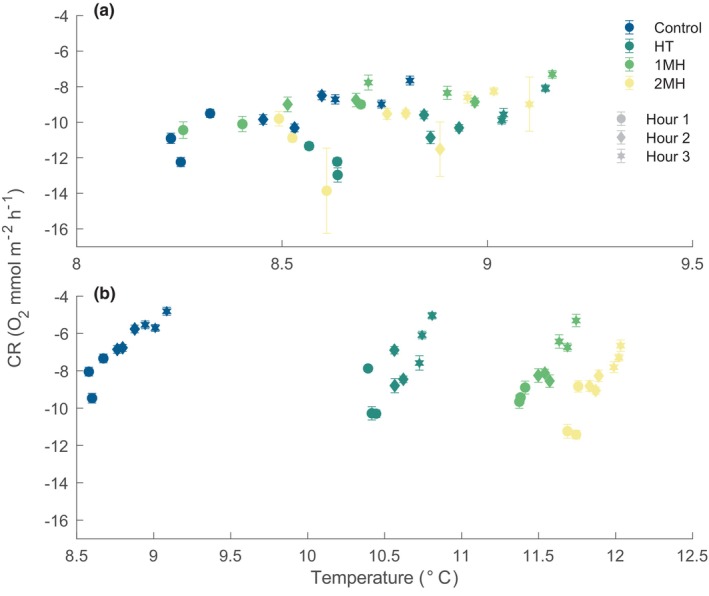
Community respiration (CR) rates during (a) dark incubation 1 (2022‐07‐01) and (b) dark incubation 2 (2022‐07‐10). Dark incubation 1 took place when all treatment conditions were receiving ambient, non‐manipulated water. Dark incubation 2 was conducted when all treatments were at setpoint conditions. Note the difference in x‐axis scale. Error bars are the SE of the linear model rate estimate.

The first community dark incubation occurred when all treatments were at ambient conditions. Temperature deviated from its target level by ~0.9°C across treatments during this incubation (Figure 4). There was a significant effect of time on CR rates between hour 1 and 3 (*p*‐value < .001), for both the 1st and 2nd dark incubation (Table S2; Figure 4). CR rates for the 2MH treatment were significantly different from the control during dark incubation 2 on 2022‐07‐10, when temperature at the start of the incubation was 11.7°C compared to 8.5°C for the control (hour 1: *p*‐value = .047, hour 2: *p*‐value = .002, hour 3: *p*‐value = .022). To note, the 1MH treatment was at a peak temperature of 11.3°C and displayed no significant difference from the control.

### 3.3 Temperature effects on net community production

**FIGURE 5:**
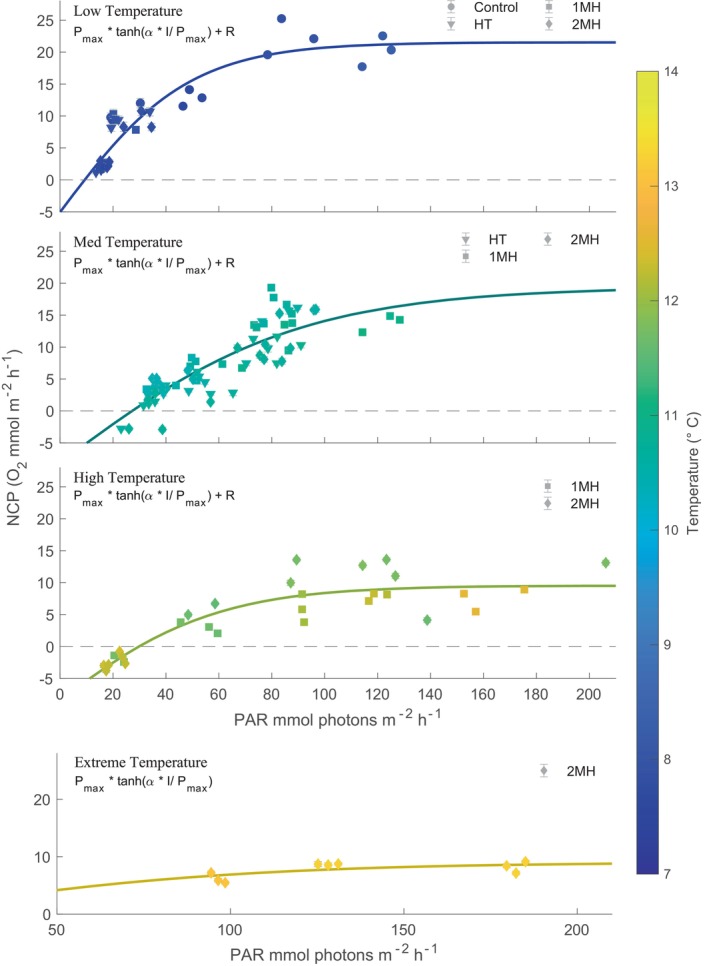
Net community production (NCP) rates separated by different temperature bins. Gray shading around the curve fits is the 95% CI of the model fit (Table 1). Error bars are the SE of the linear model rate estimate. The different treatments are: High temperature (HT), one marine heatwave (1MH), and two marine heatwaves (2MH). Note the different axis scale for the extreme temperature subplot.

**FIGURE 6:**
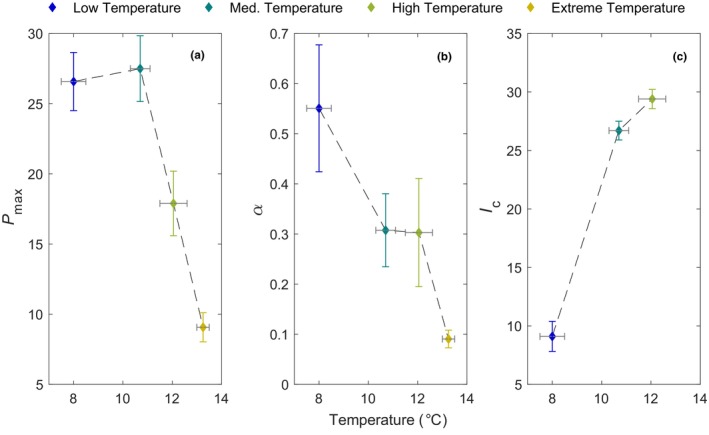
Estimated coefficients for each temperature bin scenario derived from a hyperbolic tangent model (Table 1). (a) *P*_max_ is the maximum net community production rate (mmol O_2_ m^−2^ h^−1^), (b) *α* is the photosynthetic efficiency (mmol O_2_ m^−2^ h^−1^(mmol photons m^−2^ h^−1^)^−1^) and (c) *I*_c_ is the compensation irradiance (mmol photons m^−2^ h^−1^). Horizontal error bars are the temperature range of the binned temperature scenario, and the vertical error bars are the SE of the coefficient estimate.

NCP rates decreased with increasing temperature when pooling across treatments into the four temperature scenarios (Figure 5). Model fits were robust for the low, medium, high, and extreme temperatures where the RMSE was <2.7 mmol O_2_ m^−2^ h^−1^ for all temperature scenarios (Table 1). Model predictions for the extreme temperature scenario were restricted to α and *P*_max_ due to high PAR flux at these temperatures (13.0–13.5°C), which provided a weak estimation of the *I*_c_ and respiration. Coefficient estimates for all temperature scenario models were significant for all parameters—note there is no respiration estimate for the extreme temperature scenario (Table 1). The *P*_max_ and *α* values significantly decreased for the high and extremely high temperature scenarios indicated by non‐overlapping SE estimates (Figure 6). When above 11°C, *P*_max_ decreased to below 18 mmol O_2_ m^−2^ h^−1^ compared to >25 mmol O_2_ m^−2^ h^−1^ when temperature was lower than 11°C (Figure 6). The *α* value decreased by ~50% from the lowest temperature scenario to the medium and high scenarios. For the extreme temperature scenario, there was a further decrease in *α* by >80% when compared with the lowest temperature scenario. *I*_c_ was significantly lower (non‐overlapping SE) for the low temperature scenario compared with the medium and high temperature scenarios.

### 3.4 Predicted impacts of simulated heatwaves

**FIGURE 7:**
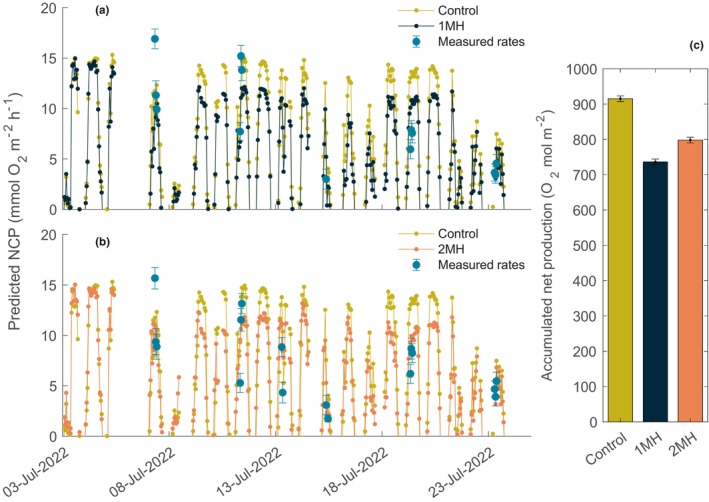
Predicted net community production (NCP) rates for the (a) one marine heatwave (1MH) and (b) two marine heatwave (2MH) treatments from the model, including the control. The average temperature and photosynthetically available radiation data from the control, 1MH, and 2MH treatments measured across the entire experimental period were used for the model inputs. Shaded color region represents the 95% CI, and the error for the measured rates is the linear model SE. Actual measured rates during incubations are shown in blue. Respiration values are not shown. (c) Accumulated NCP from all estimated rates for the control, 1MH, and 2MH treatments. Accumulated NCP does not include estimated community respiration rates. Error bars are the 95% CI calculated from a random sampling of 1000 values within the model 95% CI.

The 1MH and 2MH treatments displayed significant differences in their coefficient estimate as a predictor variable of NCP (*p*‐value = .0387). Neither treatment, however, displayed a time effect on NCP rates suggesting no difference from the beginning of the experiment to the end, after exposure to the simulated MHW scenarios. The incorporation of temperature into the hyperbolic tangent model produced a robust model fit for the *P*_max_ and α coefficients (Table 2). The impact of temperature on *P*_max_ in the model increased the saturating irradiance point (Figure S5). Respiration was fit poorly by the model; thus, accumulated net community production excluded rates predicted below zero. Model predictions of daily NCP estimates over the 3‐week period in which the experiment was conducted suggests that the 1MH treatment had a lower accumulated net production compared to the control and the 2MH treatment (Figure 7). The accumulated net community production was 736 mol O_2_ m^−2^ compared with 915 for the control, and 798 mol O_2_ m^−2^ for the 2MH treatment (Figure 7). These estimates were significant when comparing overlap of the 95% CI. Thus, the 1MH and 2MH treatments differed in accumulated net production from the control and between each other. The model predicted NCP rates were coherent to the actual measured rates, where the measured rates were on average 11% and 27% higher for the 1MH and 2MH scenarios, respectively. Although seemingly large deviations for the 1MH and 2MH treatments, 6% of the variance between the predicted and measured values was driven by two anomalous points for the 1MH, and 15% by three points for the 2MH scenario (Figure 7). This means that >80% of the predicted values were extremely coherent to actual measured NCP rates.

**TABLE 2:** Model fit and estimate from the modified hyperbolic tangent model (Equation 1) with photosynthetically available radiation PAR and temperature as predictor variables of net community production.

Parameter	Coefficients estimate	SE	*t*Stat	*p‐*Value
*P*_max_	22.00	1.055	20.86	<.001
Alpha	0.420	0.059	7.097	<.001
CR	1.418	1.148	1.235	.219
Observations (*n*)	171			
RMSE	3.79			
*F*‐statistic	106			
*p‐*Value	<0.001			

Abbreviation: CR, community respiration.

**FIGURE 8:**
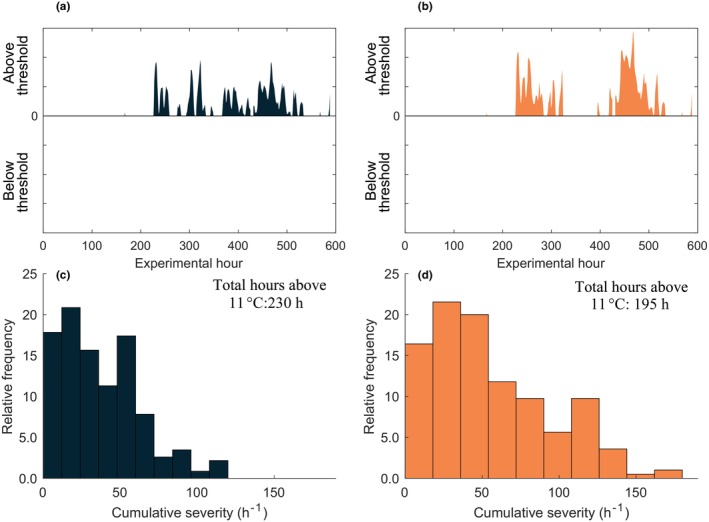
Number of hours for the (a) 1MH and (b) 2MH treatments that exceeded the 11°C threshold. (c) Cumulative severity for the 1MH and (d) 2MH treatments shown as a relative frequency (%) distribution.

The 1MH treatment which experienced an offset from the control of +2.8°C for 13 days was exposed to the longest period of cumulative temperature severity using a threshold of 11°C (Figure 8). In total, the 1MH treatment experienced 230 h of temperatures above 11°C compared with 195 h for the 2MH treatment. For both the 1MH and 2MH treatments, the cumulative severity values occurring at the highest relative frequency (~20%) was between 20 and 40 h^−1^. The 2MH treatment experienced the greatest cumulative severity values at ~150 h^−1^, but at a frequency of <1% (Figure 8). The 1MH treatment experienced ~30% more occurrences at a cumulative severity of <100 h^−1^ than the 2MH treatment.

## 4 DISCUSSION

The shallow marginal seas of the Arctic, where kelp communities thrive on rocky substrata and are expected to expand due to increased habitat availability from reduced sea‐ice coverage, will face increasing exposure to the growing frequency and intensity of MHWs (Barkhordarian et al., 2024; Krause‐Jensen et al., 2020). The results presented here show that MHWs can manifest as decreasing NCP rates, and declines in accumulated net production by mixed sporophyte kelp communities. The characteristics in how MHWs occur (i.e., its intensity and duration) will impact the degree of response by kelp communities. We found that although more extreme temperature anomalies resulted in a greater depression of NCP rates, a lower magnitude anomaly with a longer duration had a greater negative effect on the accumulated net production by the community. This was realized as a decrease in accumulated net community production by 20% for the 1MH and 13% for the 2MH scenario over a 3‐week period.

For the Barents Sea as a whole, which extends to the coasts of northern Norway and the region where this experiment took place, the annual mean frequency of MHWs has tripled over the past 20 years compared with a pre‐2004 period (Mohamed et al., 2022). Although this study reports on NCP rates and accumulated net production by mixed kelp communities, other studies have shown that an increased occurrence of MHWs on kelp communities leads to declines in biodiversity and kelp biomass when mortality temperature thresholds are surpassed for kelp species and associated community fauna (Arafeh‐Dalmau et al., 2019; Filbee‐Dexter et al., 2020; Smale et al., 2019). The results here corroborate those previous studies as both MHW treatments led to an increase in the rate of biomass loss, and a greater percent change in total biomass loss at *T*_F_ (see Appendix S1). This observation, however, is derived from a community response of mixed kelp assemblages where one species may be more resilient than another. It would be remiss to suggest that all species are negatively affected as a companion study found non‐significant differences in kelp elongation across these same treatments from a subset of individuals (Lebrun, A., Miller, C.A., Gazeau, F., Urrutti, P., Alliouane, S., Gattuso, J‐P., Comeau, S., unpublished data).

Although this study exposed kelp communities to relevant temperature anomalies for current and future temperature baselines, the maximum temperature reached by the 1MH and 2MH treatments (13.0 and 13.8°C, respectively) fall within the range of optimal or, tolerated, temperature for the species examined (Bolton & Lüning, 1982; Davison, 1987; Davison & Davison, 1987; Liesner et al., 2020). What is of importance, however, is the acclimatization potential to MHWs by specific ecotypes. In southern Norway, for example, the frequency and intensity of MHWs has been correlated to decreasing kelp biomass resulting from an increasing trend in the duration of temperature anomalies at a rate of 0.17 days year^−1^ over the past 60 years (Filbee‐Dexter et al., 2020). This leads to temperatures surpassing the mortality threshold of 19.7°C for populations of *S. latissima*, a species identified in our mesocosm experiment. During our experiment, temperatures reached a maximum of ~14°C, which should be below the mortality threshold, albeit these populations are from an Arctic fjord in northern Norway rather than southern Norway. This is particularly relevant given that ecotypes may demonstrate a difference in temperature tolerance (King et al., 2019). It is important to note that the negative effects of MHWs presented here do not indicate mass mortality or significant senesce, but more of a sublethal effect on community production. However, the sublethal effects of MHWs can drive changes in kelp community function and structure over longer time scales, as consistently high temperatures can reduce photosynthetic pigment concentration, increase respiration, and reduce overall net production (Andersen et al., 2013). This lower physiological performance not only induces tissue damage and reduced growth, but can also weaken competitiveness and facilitate turf algae growth, particular when co‐occuring with other stressors such as eutrophication (Christie, Andersen, et al., 2019; Moy & Christie, 2012; Simonson et al., 2015).

A high thermal tolerance among the kelp species studied in this experiment does not directly translate to a tolerance to short and intense warming anomalies that are MHWs. Recent evidence has shown a depression in physiological metrics (e.g., de‐epoxidation state and chlorophyll *a* concentration) for the southernmost populations (54° N) of *S. latissima* across a latitudinal study, while no effect was observed for populations further north (up to 79° N; Diehl et al., 2021). In addition, the authors suggest that a stepwise temperature increase from an absolute temperature of 16 to 18°C for the northern Norwegian coast populations, and a 10 to 12°C change for the northern most populations in Spitsbergen, may have provided a buffer period for acclimatization to short‐term exposure over 8 days. Thus, the seasonal effects of a MHW may result in a differing response, and we note that our experiment was conducted in early summer. Interestingly, *S. latissima* has been shown to potentially carry a thermal history where exposure to a previously high temperature anomaly, or its accumulated exposure duration reduced its tolerance to future anomalies (Niedzwiedz et al., 2022). It should be noted, however, that this may be a result of irreparable damage when exposed to a previous warming anomaly. The species *L. digitata* responded similarly, as heat stress exposure was better tolerated in spring than in autumn after accumulated exposure days to high temperature increased the susceptibility to heat stress (Hereward et al., 2020). These previous findings, which suggests that an accumulated exposure duration reduces tolerance, supports our findings with respect to the response of the 1MH treatment (long exposure duration) resulting in a greater decline in seasonal production. Further, the 2MH experiment tested here was a short‐term, acute stress, and may have acted as a stepwise acclimatization buffer.

The effect of temperature on kelp photosynthetic rates has been well documented (Andersen et al., 2013; Bolton & Lüning, 1982; Davison et al., 1991; Davison & Davison, 1987). The temperature threshold identified for kelp community production in this study defines a clear limit of 11°C for these lower Arctic kelp ecotypes. Light and nitrogen limitation have also been shown to modify temperature tolerance adding an additional layer of complexity when understanding the productive capacity of kelp to heat stress (Bass et al., 2023; Davison et al., 1991; Fernández et al., 2020; Niedzwiedz et al., 2022). Here, light and nutrients were similar across treatments (Figure 2 and Figure S7). An important distinction germane to the findings presented here is that ecotypes matter. For example, Diehl et al. (2021) exposed *S. latissima* tissue from sporophytes sampled from Helgoland (Germany) to Spitsbergen (Svalbard) and found that ecotypes above the Arctic circle were tolerant to a +6°C increase from natural conditions. Southern ecotypes, however, displayed physiological stress responses and tissue necrosis. Further, this high temperature tolerance appears to translate to mixed kelp assemblages in the Arctic and not just single species, as exposure to temperatures of +5.3°C from natural conditions was well tolerated by kelp populations in Kongsfjorden (Miller et al., 2024a). Even within the same region, trailing edge populations have been shown to be more thermotolerant than center populations of *L. digitata* (King et al., 2019). These findings demonstrate that within species tolerance can be broad, and that lethal and sublethal temperatures in some regions is not universal for kelp species.

The decrease in kelp NCP and accumulated net production from the exposure to the simulated MHWs may be partially explained by a decrease in chlorophyll *a* concentration and photosynthesis over time for *A. esculenta* (Lebrun, A., Miller, C.A., Gazeau, F., Urrutti, P., Alliouane, S., Gattuso, J‐P, Comeau, S., unpublished data), which represented ~40% of the total kelp biomass in each mesocosm. This reduction in photosynthetic capacity by the one species could likely explain the findings here. This would suggest, however, that *S. latissima* and *L. digitata* remained fairly tolerant to the simulated MHWs. This aligns well with our findings, as there was no significant difference found in NCP when comparing incubations two and eight, which represents the long‐term exposure effect for both MHW treatments (i.e., incubations before the induced heatwave simulation and the remission of the heatwave simulations). However, the low light levels during incubation eight make it difficult for a direct time component comparison between incubations two and eight. Additionally, the tolerance of ecotypes within a population may also play a role in the response found. The sporophytes collected in this study spanned a 44 km range and depths between 1 and 7 m. The acclimation to a specific depth could also produce variations in thermotolerance (Franke et al., 2021). While certain species may be more negatively affected by MHWs, the community response shown here supports the potential resilience of community structure despite the sublethal effect of depressed production. This is not to state that there are no negative implications for the future structure and function of kelp communities in this lower Arctic region. For certain macrophyte communities, exposure to MHWs caused alterations to the release of dissolved organic carbon impacting the carbon cycling of benthic ecosystems (Egea et al., 2023). Additionally, exposure to MHWs can modify the diversity–stability relationship within kelp communities modifying the habitat functioning of kelp forests (Liang et al., 2024). Thus, the negative effects presented here deserve further investigation as it relates to carbon cycling and the stability of community structure over longer timescales.

To provide a more comprehensive picture of the results presented here, further investigation is needed to examine the recovery period of kelp and the effects of MHWs on different life‐stages. Understanding the recovery period of kelp to warming anomalies will be imperative for determining how Arctic kelp respond to the characteristics of a MHW. Of relevance, is the exposure period. Simonson et al. (2015) reported that the difference between a 1 week and a 2–3 week exposure changed from a reduction in blade strength at 1 week, to mortality at 2–3 weeks. What remains unknown, however, is the recovery time. There is evidence to suggest that communities with a high potential for rapid recovery from disturbances may experience a tradeoff in their overall resilience to a stress (Eisenhauer et al., 2024). In this experiment, we found that NCP decreased when exposed to MHWs, but the mixed kelp assemblages appeared to recover their NCP potential rapidly. While an apparent short‐term recovery was supported in this study, what remains unclear is how this affects growth, and recruitment—both of which temperature can modify (Farrugia Drakard et al., 2023 and references therein). These are additional responses that need to be addressed as decreases in NCP—as observed here—can lead to changes in carbon cycling, net biomass gain of kelp forests, and release of detrital material (Krumhansl & Scheibling, 2012; Nardelli et al., 2024).

Despite the versatility of the experimental system to manipulate temperature and maintain flow‐through rates in each mesocosm, the true dynamics and biological interactions of an in situ community are difficult to replicate. The direct limitations of mesocosms come from “wall effects” which can change the flow regime, reflect or absorb light, and facilitate biofilm growth on the wall substrate. These random effects lend limitations to direct comparisons to real in situ conditions; however, the large volume of the mesocosms in this study, the automated flow‐through system, and cleaning of mesocosm walls likely limited these biases. Additionally, the relative difference between treatments will not change the response observed to the manipulated temperature effect.

## 5 CONCLUSION

We have demonstrated that the environmental conditions of a future climate with more intense MHWs produces sublethal effects expressed as decreased NCP and accumulated net production. Kelp communities exposed to two short‐term MHWs showed no indication of a delayed negative response with respect to NCP, whereas exposure to one long‐term MHW appeared to have a greater negative impact and cumulative severity. Although the findings presented here cannot conclude on the lethal effects MHWs have on kelp sporophytes, the importance of sublethal effects are acknowledged. This study provides crucial insight into how MHWs can modify kelp community production and, thus, the potential structure and function of these benthic biogenic habitats.

## AUTHOR CONTRIBUTIONS

**Cale A. Miller:** Conceptualization (equal); data curation (equal); formal analysis (lead); methodology (equal); writing – original draft (lead). **Frédéric Gazeau:** Conceptualization (equal); investigation (equal); methodology (equal); writing – review and editing (equal). **Anaïs Lebrun:** Conceptualization (equal); data curation (equal); investigation (equal). **Samir Alliouane:** Data curation (equal); investigation (equal). **Pierre Urrutti:** Conceptualization (equal); methodology (equal). **Robert W. Schlegel:** Conceptualization (equal); resources (equal); writing – review and editing (equal). **Jean‐Pierre Gattuso:** Conceptualization (equal); funding acquisition (equal); investigation (equal); writing – review and editing (equal). **Steeve Comeau:** Conceptualization (equal); data curation (equal); funding acquisition (equal); methodology (equal); writing – review and editing (equal).

## CONFLICT OF INTEREST STATEMENT

The authors have no competing interests to declare.

### OPEN RESEARCH BADGES

This article has earned an Open Data badge for making publicly available the digitally‐shareable data necessary to reproduce the reported results. The data is available at https://doi.org/10.1594/PANGAEA.967060.

## Supporting information


Appendix S1

## Data Availability

Data supporting this study are openly available on the PANGAEA World data center at https://doi.org/10.1594/PANGAEA.967060: Miller et al. (2024b).
