# Supplementary material for: Summer primary production of Arctic kelp communities is more affected by duration than magnitude of simulated marine heatwaves
Source: Ecol Evol. 2024 Sep 29;14(10):e70183. doi: 10.1002/ece3.70183 (PMC11439588; doi:10.1002/ece3.70183)
Supplement: Supplementary file 1 — Appendix S1 [file ECE3-14-e70183-s001.docx]

**Supplementary material**

**Table S1**. Linear stepwise model results comparing treatments.

**Table S2**. Repeated measure ANOVA table comparing within treatment differences for dark respiration incubations.

**Figure S1.** Median temperature at three different locations in the Troms region. The mid latitude of 69.875°N (red line) corresponds to the collection sites of organisms used for this experiment. Temperature values were collected from the NOAA Optimum Interpolation Sea Surface Temperature (OISST) daily v2.1 satellite dataset.

**Figure S2**. Mean temperature and salinity for each treatment over the entire experimental period.

**Figure S3**. Boxplots of initial and final fresh weight (fw) biomass. No significant difference in initial biomass (*p*-value = 0.187). Final biomass was significantly different for 1MH (*p*-value = 0.0171).

**Figure S4.** Total kelp fresh weight (fw) biomass at the beginning (T_0_) of the experiment and at the end (T_F_). Each line represents one of the three replicates for reach treatment: replicate 1 is blue, replicate 2 is red, and replicate 3 is yellow.

**Figure S5**. Predictor effects: partial dependence models of PAR (mmol photons m^-2^ h^-1^) and

temperature (°C).

**Figure S5**. Probability distribution of the 95% CI from a Monte Carlo simulation for the control, 1MH, and 2MH model estimate of total accumulated net production. The area under the curve (AUC) value is the CI estimate.

Nutrient samples were collected before and directly after an incubation using a handheld syringe with a 0.22 μm cellulose acetate filter. Samples were collected in an acid washed 60 ml HDPE (high-density polyethylene) bottle and immediately frozen at -20°C. All samples were analyzed on an AA3 170 autoanalyzer (SEAL Analytical) at the Laboritore d’Oceanographie de Villefranche. Not all incubation periods were sampled for nutrients due to logistical constraints of sample transport. Significant differences between T0 and TF uptake rates were only observed during incubation 8 for N+N in the 2MH treatment.


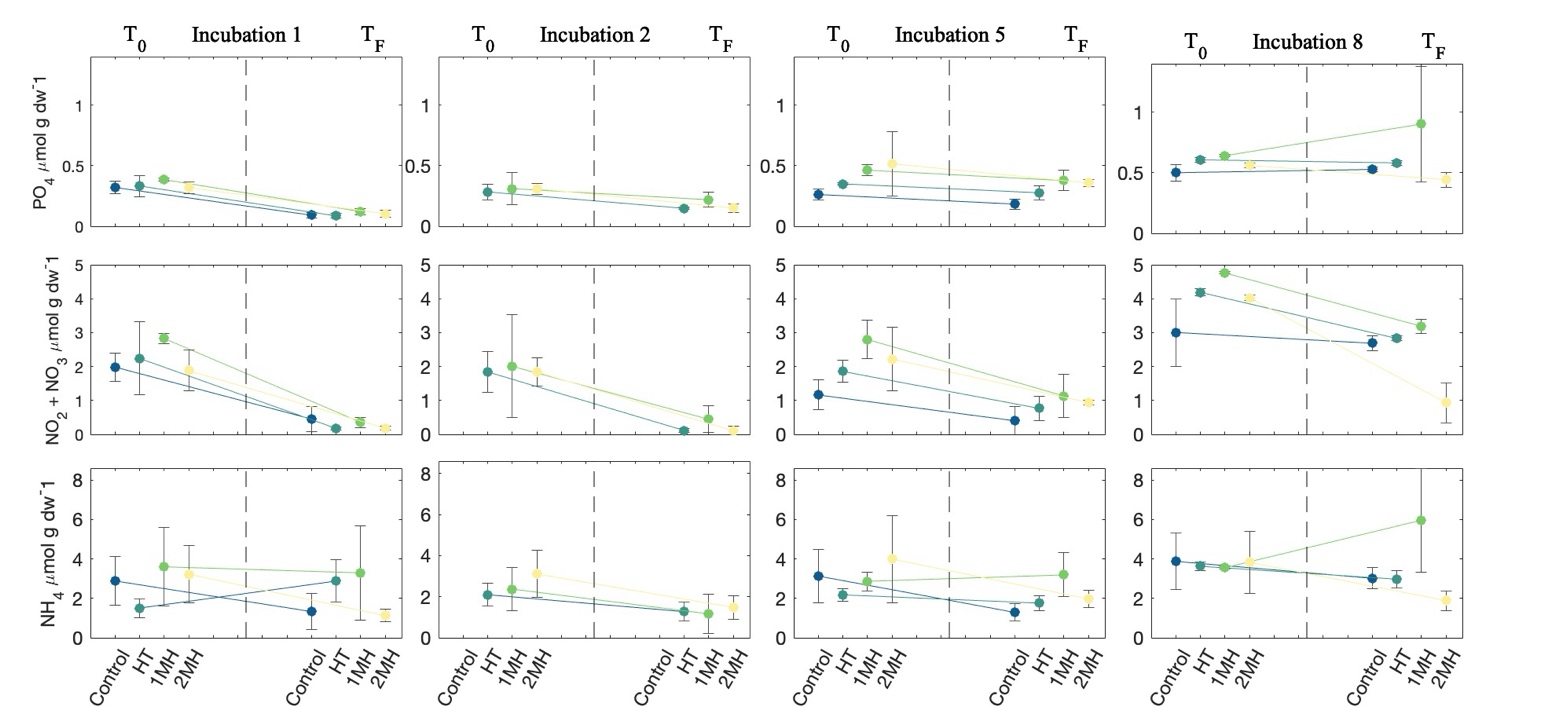


**Figure S7**. Nutrient data for incubations 1, 2, 5, and 8 were blue is the control, blue-green the HT treatment, green the 1MH treatment, and yellow the 2MH treatment. Error bars are the SD.
